# “It’s a delicate dance”: How informal caregivers experience the role and responsibilities of supporting someone living with a lower-grade glioma

**DOI:** 10.1093/nop/npae096

**Published:** 2024-10-14

**Authors:** Ben Rimmer, Michelle Balla, Lizzie Dutton, Richéal Burns, Vera Araújo-Soares, Tracy Finch, Joanne Lewis, Pamela Gallagher, Sophie Williams, Fiona Menger, Linda Sharp

**Affiliations:** Population Health Sciences Institute, Newcastle University, Newcastle University Centre for Cancer, Newcastle upon Tyne, England; Faculty of Medical Sciences, Newcastle University, Newcastle upon Tyne, England; Population Health Sciences Institute, Newcastle University, Newcastle University Centre for Cancer, Newcastle upon Tyne, England; Faculty of Science, Atlantic Technological University, Sligo, Ireland; Health and Biomedical Strategic Research Centre, Atlantic Technological University, Sligo, Ireland; Population Health Sciences Institute, Newcastle University, Newcastle University Centre for Cancer, Newcastle upon Tyne, England; Centre for Preventive Medicine and Digital Health, Department for Prevention of Cardiovascular and Metabolic Disease, Medical Faculty Mannheim, Heidelberg University, Heidelberg, Germany; Department of Nursing, Midwifery and Health, Northumbria University, Newcastle upon Tyne, England; Newcastle upon Tyne Hospitals NHS Foundation Trust, Newcastle upon Tyne, England; School of Psychology, Dublin City University, Dublin, Ireland; Newcastle upon Tyne Hospitals NHS Foundation Trust, Newcastle upon Tyne, England; School of Education, Communication and Language Sciences, Newcastle University, Newcastle upon Tyne, England; Population Health Sciences Institute, Newcastle University, Newcastle University Centre for Cancer, Newcastle upon Tyne, England

**Keywords:** informal caregiving, role, responsibilities, lower-grade glioma, qualitative

## Abstract

**Background:**

People with lower-grade gliomas (LGG) often require long-term support with a condition that causes substantial symptom burden and is likely to progress. Partners, family, and friends often become informal caregivers (IC), but the types of support they provide, and their experiences of this, have not been well investigated. We aimed to understand how ICs experience the role and responsibilities of supporting people with LGG.

**Methods:**

This descriptive qualitative study used semistructured interviews to explore the role and responsibilities of a purposive sample of ICs across the United Kingdom, who currently, or in the past 5 years, support(ed) someone with an LGG. Interviews were audio-recorded and transcribed, and an inductive thematic analysis was conducted.

**Results:**

Nineteen ICs were interviewed (mean age 54.6 years; 5 males/14 females). While most participants spoke about “Being a ‘carer’,” the level of care provided varied. Participants conveyed their experiences with “Adjusting for cognitive difficulties,” “Emotional protection,” “Supporting participation in daily life,” and “Healthcare advocacy.” ICs often felt “abandoned” by healthcare services to provide required care themselves, and reported experiences with “Balancing the challenges of caregiving,” including conflict with work/childcare. Issues around “Maintaining the care recipient’s independence” were interwoven throughout.

**Conclusions:**

ICs of people with LGG provide wide-ranging support to help manage the consequences of the illness. Consideration of ways to help ICs with the challenges of fulfilling this role, particularly, balancing support provision without inhibiting the care recipient’s independence, could help improve outcomes for ICs and people with LGG.

Lower-grade gliomas (LGG; eg, grade 2 astrocytoma and grade 2 or 3 oligodendroglioma^1^) are largely diagnosed in adults in their 30s and 40s,^2^ and account for approximately 15% of all gliomas, which are the most common group of malignant brain tumors.^3^ LGGs are incurable and will progress to high-grade glioma (HGG), limiting life expectancy to 5-15 years following diagnosis, with shorter progression-free survival in people with grade 2 astrocytomas, compared to oligodendrogliomas.^3,4^ People with LGG often experience changes in social roles, functions of everyday living, and loss of independence,^5^ as a result of numerous, often co-occurring, symptoms and impairments (eg, fatigue, seizures, cognitive deficits, personality changes, and mobility issues)^6^ arising from/consequent to the tumor and its treatment. This can have a profound impact on both the individuals, and their family, with the impact exacerbated by the relatively long-term prognosis.

Family members and friends, particularly partners of people living with and beyond cancer, often adopt the role of informal caregiver (IC), which pertains to the provision of ongoing support and care, without pay.^7^ ICs have an integral role in helping the care recipient to manage the consequences of their illness^8^; in studies in other cancers, the support provided typically encompassed emotional (eg, maintaining positivity)^7,9^ and practical support (eg, housework, transport, and finances),^10,11^ as well as assisting with healthcare decision-making.^9,12,13^ The weight of responsibility of fulfilling the role and responsibilities of being an IC is often unrecognized.^14,15^

There is a growing evidence base of studies that have investigated the experiences of ICs who support someone with a brain tumor.^16,17^ While these studies suggest some similarities with the support provided by ICs of other cancers,^18,19^ people with LGG may also have similar caregiving needs to people with acquired brain injuries (eg, cognitive support).^20^ Indeed, the brain tumor literature highlights the importance of, and need for, cognitive support (eg, strategies to facilitate memory),^21,22^ due to potential cognitive impairments.^23^ However, existing studies largely include samples of ICs in support of people with HGG who, typically, have a much shorter prognosis and higher physical dependency than those diagnosed with an LGG.^19,21^ Only one study appears to have reported any data on the support role of ICs for people with LGG,^24^ though their focus concerned the impact of being an IC, rather than ICs’ experiences of providing different types of support. A better understanding of these experiences is important for considering whether ICs feel able to provide the support required by people with LGG and what support ICs themselves might need to fulfill this role. Consequently, this study aimed to understand how ICs experience the role and responsibilities of supporting someone with an LGG.

## Methods

### Design

This qualitative study, part of the wider multi-method Ways Ahead project,^25^ used semistructured interviews to generate data on the lived experiences of being an IC for someone with an LGG, primarily to understand how ICs experience the role and responsibilities of supporting people with LGG. As this is an area where little is known, this study was descriptive in design to recognize, and facilitate exploration of, the subjective and diverse nature of participants’ experiences.^26^ Due to the richness of the collected data, we have reported elsewhere on the emotional impact of being an IC for someone with an LGG^27^; the 2 papers are thus complementary. Ways Ahead was reviewed and approved by the Wales Research Ethics Committee (REC ref: 20/WA/0118).

### Participants and Recruitment

Individuals were eligible if they lived in the United Kingdom, were aged 18 years or older, and were family members or friends who identified themselves as currently supporting, or having supported in the past 5 years, someone with an LGG (defined, in this study, as someone with a grade 2 astrocytoma or grade 2 or 3 oligodendroglioma).^1^ Individuals who were bereaved at the time of recruitment, but were a caregiver in the past 5 years, were considered eligible. Purposive sampling was used to recruit a range of ages, sex, and relationships to the care recipient.

Recruitment occurred through one of 2 avenues: (1) healthcare professionals at collaborating National Health Service (NHS) sites provided potentially eligible individuals with an information sheet; (2) the study was advertised through the Brain Tumour Charity, with the information sheet attached. Individuals were approached, using the terms “family-member or friend” (rather than “carer”). The information sheet provided a brief introduction to the study and the researchers conducting the interviews. To register interest, individuals were asked to call or email the study team. The researchers (B.R. and L.D.) subsequently telephoned each individual to confirm eligibility and afford the opportunity to ask questions; if they were eligible and willing to take part, a convenient date and time for the interview was arranged. Participants were recruited between August 2020 and March 2022. Twenty-two of the 24 ICs that registered an interest in taking part were eligible; for the other 2, the care recipients did not have an LGG. Three people did not respond to attempts to schedule an interview.

### Data Collection

Trained and experienced in qualitative research, B.R. (male, MSc, Research Assistant) and L.D. (female, PhD, Research Associate) conducted the interviews. As per interviewee preference, remote interviews were conducted using video-conferencing software (eg, Zoom) or telephone. Immediately prior to the interview, we acquired audio-recorded consent from all participants and collected basic demographics (eg, age, sex, employment, and relationship status).

Semistructured interviews followed a topic guide (Supplementary File 1) that was initially informed by the brain tumor caregiving literature, and modified following discussion with a patient and public involvement (PPI) panel, which included people with brain tumors (*n* = 3) and ICs of people with brain tumors (*n* = 3); members of the PPI panel did not take part in the study. The guide comprised open questions and was used flexibly, depending on what the participant spoke about and the order in which they discussed the issues. Any new issues raised throughout data collection were added to the topic guide to be explored in subsequent interviews.

We asked participants to broadly reflect on their experiences of supporting someone with an LGG. We also explored the interviewee’s perceptions of how they and their care recipient had been impacted by the illness and consequent support needs across various areas (eg, emotions, relationships, and transport). We asked about the responsibilities and challenges involved in supporting the care recipient, as well as what, and when, (in)formal support was received or needed by the IC to help them fulfill their caring responsibilities. We used probing questions, where appropriate, to explore areas further. Throughout each interview, participants were encouraged to think beyond the period immediately following the initial diagnosis. There were opportunities during the interview for participants to raise any additional issues they wished to discuss. To finish, a postinterview sheet with details of charities and helplines was provided, and as a thank you, we offered participants a £20 voucher. Each interview was audio-recorded and lasted on average 85 minutes (range 54-110 minutes). During each interview, the researcher took field notes for their own reference.

### Data Analysis

Interviews were transcribed verbatim and anonymized. We conducted an inductive thematic analysis^28^ on the entire dataset, and report here data *specifically* related to the role and responsibilities of ICs. This approach to analysis was chosen for its ability to develop data-driven patterns of meaning, and therefore, help understand how ICs might experience their role and responsibilities in different ways.

We took several steps to ensure rigor (eg, credibility, dependability, and confirmability)^29^ throughout data analysis: (1) We conducted data collection and analysis in parallel to ensure that any new issues raised were explored in subsequent interviews. (2) Following familiarization with the data, B.R. and M.B. (both trained in qualitative research) independently generated initial codes, using NVivo, for a sample of transcripts (*n* = 5 of 19). (3) B.R. and M.B. discussed preliminary codes to create a combined code list; B.R. applied this to the remaining transcripts, adding any new codes as the analysis progressed (and also returning to annotate earlier transcripts with these new codes). Codes and uncertainties were regularly discussed within the research team as this process progressed. (4) B.R. organized these codes to construct preliminary themes at the semantic level; these themes were modified and refined following discussion with the wider analysis team (M..B and L.S.). (5) We ceased recruitment once data sufficiency occurred; this was determined by the researchers’ judgment that there was sufficient data to understand ICs’ lived experiences of supporting people with LGG, and specifically for this paper, how ICs experience the role and responsibilities of supporting someone with an LGG.^30^ Each participant was given a summary of findings and afforded the opportunity to provide feedback. The finalized themes are reported below.

## Results

### Participant Characteristics

Nineteen ICs were interviewed (7 recruited through NHS sites; 12 through the Brain Tumour Charity). Fourteen participants were female; at the interview, mean age of all participants was 54.6 years (range 36-78 years), and 13 were employed (Table 1). All except one participant was married. Fifteen participants were spouses, 2 were sisters, and 2 mothers of people with LGG. Fifteen participants lived in the same household as the care recipient. Six participants (all spouses) had children aged <18 years. None of the participants were bereaved.

**Table 1.** Informal caregiver participant characteristics (*n* = 19)

Characteristic	*n*	Characteristic	Mean (range)
*Sex*		*Full-time education (years)*	14.9 (10-18)
Female	14	*Relationship to care recipient*	*n*
Male	5	Wife	10
*Age*		Husband	5
≤ 40	3	Mother	2
41-50	3	Sister	2
51-60	8	*Relationship status*	
> 60	5	Married	18
*Employment status*		Single	1
Full-time employee	10	*Dependents*	
Part-time employee	3	None	13
Retired	4	One	3
Caring for family	2	Two	3
*Co-habiting with the care recipient?*			
Yes	15		
No	4		

### Overview of Findings

**Figure 1.**
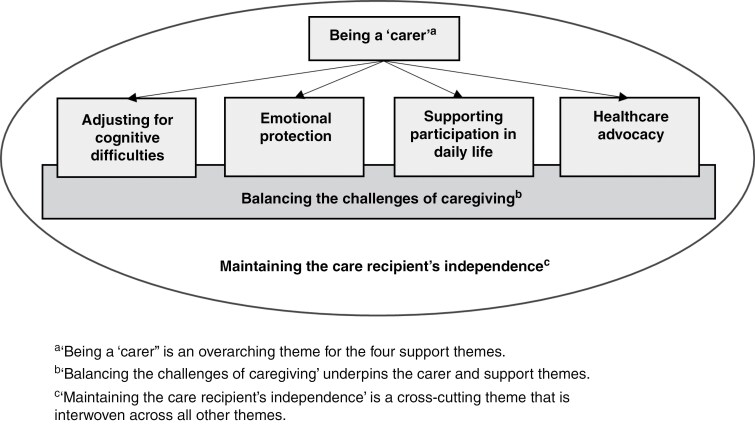
Overview of themes for the role and responsibilities of being an IC for someone with an LGG

We constructed 7 themes, shown in Figure 1 with supporting quotes in Table 2, accompanied by the IC’s age at the interview and relationship to the care recipient. While most participants perceived themselves as having assumed a caring role, the level of care provided varied (overarching theme “Being a ‘carer’”). Participants reported their experiences with specific responsibilities, which encompassed “Adjusting for cognitive difficulties,” “Emotional protection,” “Supporting participation in daily life” and “Healthcare advocacy.” Underpinning the support themes, participants described experiences with “Balancing the challenges of caregiving,” which influenced their ability to fulfill their caring role and responsibilities. Participants’ attempts to find a balance between providing care and “Maintaining the care recipient’s independence” were interwoven across all themes.

**Table 2.** Supporting quotes for all themes, with participant ID, age at interview, and relationship to care recipient

Theme	Illustrative quotes
Being a “carer”	“When I use the word ‘carer’, I don’t mean that I’m looking after his every domestic need. That is not the case. He’s very independent...I feel a greater weight of responsibility about how he is, where he is, is he okay.”—IC2 (aged 55, female spouse) “I had to change my expectations because you expect him to help you out around the house and stuff. And I had to change my expectation of, right, for him to have to the energy… I need to do all the housework.”—IC15 (aged 44, female spouse) “Pretty much overnight, our relationship had to change, where it went from a partnership to me being [care recipient]’s carer.”—IC1 (aged 38, female spouse) “We’re always there if she wants us. We will always be there. We did stay while she was in the hospital. We stayed with her husband in [partner]’s home. And it’s just a case of being there really.”—IC3 (aged 78, mother) “I went into part time work, I had the flexibility then to ask if I could work this day, that day or those hours. That enabled me to keep myself at work so I was happy doing that but it also allowed me to feel that I was supporting [care recipient] to the extent he needed it.”—IC12 (aged 66, female spouse)
Adjusting for cognitive difficulties	“He’s got a diary. So, when I want him to remember things, I write in that diary. So, he looks at that diary.”—IC4 (aged 57, sister) “I have a backup alarm set in case she’s asleep and misses her Keppra [seizure medication].”—IC23 (aged 56, male spouse) “We have, like, index cards all around our house, or just little visual prompts to remind him to do things, like you know, ‘Don’t forget to turn this off’, ‘Take the plug out’, ‘Check your pockets’, ‘Your keys are on the hook’.”—IC1 (aged 38, female spouse) “She had the dosette box and then eventually she started, she couldn’t remember and so therefore we were giving her everything at set times and making sure you were giving them.”—IC8 (aged 68, mother) “Things take him longer to think through, longer to put his decisions into action. He quite often will need more confirmation from me around what he’s thinking on decisions than ever he would have done before.”—IC2 (aged 55, female spouse) “I packed the house up all by myself up north, and I had to make all the decisions regarding where we were going to live, come down and look at places…all the paperwork and everything, all the decision–making, I’ve got to make now.”—IC10 (aged 59, female spouse)
	“When fatigue kicks in, word finding can be challenging but I know not to finish sentences unless she asks me to.”—IC23 (aged 56, male spouse)
Emotional protection	“They need to be understood and really listened to and heard to make them feel not less of a person…I think that’s the most important thing, that dignity and self-esteem, about being able to preserve that.”—IC23 (aged 56, male spouse) “We are trying to get him to listen to audiobooks, those types of things, just to give him things that are a bit more relaxing, not as strenuous, things that don’t agitate him as much.”—IC1 (aged 38, female spouse) “For a very long time I’ve been very careful about putting him under any pressure. He was very stressed when he had his seizure. I’m adamant it was caused by the extreme stress.”—IC7 (aged 53, female spouse) “I think probably it’s just keeping cheerful. I mean keeping cheerful with her. I make sure that I smile…and joke with her.”—IC24 (aged 67, male spouse) “Sometimes I need time to put my feelings out. Even though I’ve been told to do this in front of [care recipient], I try not to…mainly because my concern is that showing her how I feel could create her a sense that the situation is really bad.”—IC21 (aged 36, male spouse) “It tends to take the shine off things, when something, good news, it might be the family, and he’s negative about things. I’ve tried to say to him, ‘Well, this is a positive thing, this is good!’ you know. ‘Oh, is it?’. You’ve also to try and get them not to go in on themselves”—IC10 (aged 59, female spouse)
	“I think just trying to get on and do things that we enjoy doing which sounds simple but we know what we like doing so we try and do that. We do go out and be in nature and just avoid things that stress us out and just try and do things that make us happy.”—IC19 (aged 54, female spouse)
Supporting participation in daily life	“I’ve always prioritized if [care recipient] wants to work because it makes him feel better, I’m going to make sure that he can work. In order for him to work, I have to do all the other stuff. So I fit jobs in around the hours that the boys are at school.”—IC18 (aged 48, female spouse) “We try to put the good energy that he does have into quality time with me and the kids and doing the family stuff.”—IC15 (aged 44, female spouse) “I got a bus pass for her and with that bus pass I could use it as well because she couldn’t really get on or off by herself.”—IC24 (aged 67, male spouse) “We have specific [walking] routes depending how she feels on the day. So if she’s not feeling 100% energetic we try to go to the shortest flat ones…we have to really plan this.”—IC21 (aged 36, male spouse)
	“I was staying down to offer support and care and to pick the children up from school and meet them, cook dinner.” IC8 (aged 68, mother) “I’ve got to either drive her or take her to things because she would have done it herself. I’m away to meet a pal for a coffee, I’m away to meet a work colleague...I’d rather just drive her there and you tend to join in.”—IC22 (aged 57, male spouse)
	“We had to fight for the critical illness insurance because part of his symptom was that he wasn’t opening any mail and he didn’t pay the bills.”—IC18 (aged 48, female spouse) “There’s spare cash if you want to either put some in the savings or do you want to buy a pair of trainers?”…it’s just really trying to help him to think this money needs to be used for something before you buy something else. I think if he was on his own, he would be in a dire situation right now.”—IC14 (aged 37, female spouse) “I feel like we’ve been very lucky because partly my job is sufficiently well paid that it was never going to be an impossible situation if [care recipient] couldn’t carry on working.”—IC13 (aged 51, male spouse)
Healthcare advocacy	“I find I have to kind of go along, so I can [tell them what’s wrong]… because [care recipient] would just tell them everything’s fine.”—IC15 (aged 44, female spouse) “I try and watch her, I pay a lot of attention to her, just to see, because I get asked these questions with [doctor’s name] and the like, if there’s change a lot. So, I do try and pay attention.”—IC22 (aged 57, male spouse) “I looked up things a little bit and familiarized myself with things… a little bit of knowledge has helped us to think, ‘Well that’s okay. It’s probably this,’ or, ‘When we go and see such and such person, it might be helpful if you ask them about that,’ only doing that as a way to try and reassure him.”—IC12 (aged 66, female spouse) “I try to do a lot of learning about how his brain might be working. I would say my role is definitely patient advocate for [care recipient].”—IC18 (aged 48, female spouse) “I did everything. I went on courses. I read about organic food. I went on brain tumor conferences. I just wanted to know everything.”—IC7 (aged 53, female spouse) “We also go to a specialist day center for brain injury…It took us a lot of working to get him into it. But when they met [person with LGG] and listened to me, there really was no question [about whether he should be there].”—IC1 (aged 38, female spouse) “I also have to ensure that I’ve got them [appointment dates] because I suppose that’s my control bit, isn’t it? That’s not to do with [care recipient] or the hospital, that’s me. I need to know when it is so I’ve got it and I can work my work around it. But yes, I feel I need to know it so that we don’t forget.”—IC2 (aged 55, female spouse) “I actually pushed, from day one, he did get some physio but, obviously, you only get so much physio, then it ends. But she got him a wheelchair, but then I said that it was too heavy for me to pick it up and put it in the car. So she got a lightweight one, and that was a very quick phone call.”—IC10 (aged 59, female spouse)
Balancing the challenges of caregiving	“A lot of the time it was quite difficult to have the conversations that you needed to have with even the medical staff because the children were there when they came home from school and you’d have a late afternoon phone call or something like that. It was quite difficult to have those conversations.”—IC8 (aged 68, mother) “You’re just being led by them. I feel like if they’re okay, I’m okay. If she’s happy with what’s going on, then I’m happy with what’s going on. You sort of have to put them at the forefront”—IC6 (aged 50, sister) “I needed to balance… I needed to put back what I wanted. I wanted to be there, to stay there, and to be the one taking her a cup of tea up, or whatever. But I had to accept that husbands come first.”—IC3 (aged 78, mother) “There isn’t really that support there. Because who is going to take a 40-year-old man or a 12-year-old off your hands who has these medical needs or these disabilities?... it’s having that understanding that…there are disabilities in my house. But you do feel like you are certainly abandoned just to care by yourself.”—IC1 (aged 38, female spouse) “I’ve had to do a lot of Googling myself to find out the dynamics of the tumor, what’s going to happen, how it grows. I just don’t think there’s information out there even for… if a family member wanted to look up information, there’s nothing.”—IC14 (aged 37, female spouse) “I had no idea what to expect as a consequence of the treatment. I just think, ‘Gosh, what if we hadn’t have said about that?’ because it was only in passing.”—IC2 (aged 55, female spouse) “I’ve tried other things to help him remember but he doesn’t really want to do it, like having a little diary with things written down for him but he doesn’t really want to get involved.”—IC19 (aged 54, female spouse)
Maintaining the care recipient’s independence	“It’s a delicate dance, it’s about me not doing too much for her. It’s about communicating and saying, ‘I’m going to put the laundry in unless you would like to do that today?’”—IC23 (aged 56, male spouse) “The condition isn’t physically yours it belongs to them, so you have to be led by them. As hard as that is sometimes, you have to be led by them and put yourself in their shoes... if they don’t want all the fussing, you have to respect that.”—IC6 (aged 50, sister) “Even though she knew that things [her functioning] were going, she still wanted that independence and that movement and I didn’t want to take that away from her. I wanted to try and help her more but she was determined.”—IC8 (aged 68, mother) “I feel like we’ve tried to carry on as normal, I think because [care recipient] has not been massively affected in terms of her personality and physical capability, that seems to have been manageable.”—IC13 (aged 51, male spouse) “I’ve had to try and be really strict and not remind him of things and just say, ‘Look at your calendar,’ but it’s very hard to communicate.”—IC7 (aged 53, female spouse) “That is a hard thing because when you’ve been someone’s carer and used to making all the decisions, when they get back to strength and they start making their own decisions, it’s like, “Oh okay. I have to take a back seat now.”—IC7 (aged 53, female spouse) “I try and give him independence. He does small routes with the dog. He’s got little routes that he does… I just want to care for him as best I can but still allow him that degree of independence.”—IC14 (aged 37, female spouse)

### Being a “Carer”

In this theme, participants outlined their role as a “carer” and the level of care they provide to help the care recipient to manage their illness. Many participants reported a shift in their relationship dynamic with the care recipient; this involved a change from being in a partnership with the care recipient to taking on a greater weight of responsibility as a “carer.” However, this shift did not necessarily mean the care recipient needed direct care or lacked independence, rather the IC began to feel a greater general concern for their well-being.

The level of care required by the person with LGG, and what was needed to facilitate that, varied across participants. For some ICs, it was important that they were physically close to the care recipient as often as possible to feel that they could be there for them. Other participants described the need to adjust their expectations of what was their responsibility, and what was the care recipient’s responsibility (eg, them doing more of the housework). A few participants reported changes in their occupational roles (eg, going part-time) to allow them to maintain employment while feeling able to provide the care required.

### Adjusting for Cognitive Difficulties

This theme encompassed the reported (need for) adjustment for impairments in the care recipient’s memory, communication, and executive function. Most participants reported implementing strategies to facilitate the care recipient’s memory; in relation to some things to be remembered (eg, social events, safety when cooking), participants were mindful to encourage or maintain the independence of the care recipient, by using prompts and reminders (eg, index cards, diary). However, for medication management, some ICs described how they felt the need to take more control (eg, setting their own alarms) to minimize the risk of missed medication and ensure, for example, the management of the care recipient’s seizures. Several participants reported feeling the need to take a greater role in decision-making. How ICs aided decision-making varied; some ICs provided the care recipient with reassurance that a decision they (the care recipient) had made was reasonable or could be put into action. Others reported making larger decisions (eg, moving house) on the care recipient’s behalf. A few participants detailed how the care recipient could struggle to communicate, especially when experiencing fatigue. In those instances, ICs described being patient and understanding with word-finding difficulties, giving the care recipient time to find the words.

### Emotional Protection

In this theme, most participants reported efforts to protect the care recipient’s emotional well-being, particularly related to managing the psychological consequences of living with an incurable illness. Most participants detailed the ways they provided companionship to the care recipient; this included simply “being there” for the care recipient, but also maintaining modified engagement in enjoyable activities (eg, nature walks). Several participants reported the need to acknowledge and respect the care recipient’s desired level of emotional support. They explained how they perceived that it was important to ensure that the care recipient felt listened to, and were not considered “less of a person” because of their limitations, or placed in a position where they felt overwhelmed from too much “fussing.”

Some participants reported attempts to help the care recipient maintain a positive outlook, through open communication and reassurance to “try and get them to not go in on themselves.” Some ICs reported the importance of affording the care recipient time and space to de-stress and relax when needed; sometimes they also arranged relaxing activities (eg, organizing audio books). A few ICs spoke about how they actively tried to avoid expressing negative emotions, or putting any pressure on the care recipient, to minimize the possibility of the care recipient becoming distressed; high-stress levels were believed by some ICs to be linked to seizure activity.

### Supporting Participation in Daily Life

This theme encompassed the ways that participants described supporting the care recipient’s participation in daily life, such as helping them to maintain social and occupational roles. Several participants reported how they sought to relieve the care recipient’s responsibilities by assuming a greater role with, or arranging support for, childcare and housework (eg, cooking and cleaning). ICs detailed the importance of this for enabling the care recipient to channel their energy into work or “quality time.”

Most participants reported providing support with transport; many described doing more driving to facilitate attendance to health appointments, work, or social activities, particularly while the care recipient’s driving license was revoked due to the risk of seizures. Some participants acquired resources for the care recipient (eg, bus pass) to make it easier for them to use public transport; these ICs reported often still accompanying the care recipient, as cognitive impairments impacted journey planning. Many participants reported implementing a variety of practical strategies to manage the care recipient’s challenges with fatigue and mobility (eg, risk of falls). This included adjustments to facilitate physical activity or continued engagement with hobbies (eg, taking breaks; planning shorter, manageable walking routes; and glamping instead of camping).

Several participants reported taking on more responsibility for household financial management, for example, ensuring prompt bill payments. In order to alleviate financial pressure, some ICs described a desire or pressure to earn sufficient income so that the care recipient did not need to work. Other participants encouraged the care recipient to budget and supported the maintenance of autonomy with financial management. A few participants described the challenges they experienced with convincing decision-makers that the care recipient’s symptoms and impairments were severe enough to warrant financial support (eg, critical illness insurance, personal independence payments through the social welfare system).

### Healthcare Advocacy

Most participants reported being an advocate for the care recipient’s healthcare; this theme encompassed how this was approached. This included accompanying the care recipient to health appointments; a few suggested that this ensured well-being concerns (eg, fatigue and medication side-effects) were mentioned to the care team, as otherwise, the care recipient may “tell [the healthcare professional] everything’s fine.” Some ICs described collaborating with healthcare professionals and informing them when they noticed any changes in the care recipient’s symptoms and functioning.

To equip themselves with the knowledge required to better advocate for the care recipient, the majority of ICs reported asking questions at health appointments, attending information events, or searching online. This included obtaining information about the care recipient’s diagnosis, potential prognosis, symptoms and impairments, and strategies that could help the care recipient manage life with an LGG. For some ICs, this helped them feel able to reassure the care recipient about what is “normal” and inform what questions might be useful to ask the healthcare professionals in an appointment.

Due to the perceived importance of health appointments, particularly scan appointments, some participants described taking control to ensure follow-up appointments were scheduled and, once scheduled, were not forgotten. Many participants reported seeking and arranging support, particularly from allied health professionals or support services (eg, counseling and physiotherapy) on the care recipient’s behalf; some highlighted that accessing support sometimes took a lot of perseverance.

### Balancing the Challenges of Caregiving

In this theme, most participants described wide-ranging challenges that impacted their ability to fulfill their caregiving role and responsibilities; this theme, therefore, underpinned the other support themes. Several ICs reported balancing the conflict between providing support and maintaining childcare or employment, particularly in relation to healthcare involvement (eg, due to the timing of healthcare appointments). For some participants, this extended to a perceived need to put others’ (eg, care recipient and children) needs before their own. Non-spousal ICs reported how not living with the care recipient impacted their ability to provide support. A few ICs spoke about feeling “abandoned” by the health services and a lack of formal support to provide respite for them, and described how this exacerbated the challenges they faced. Some participants perceived a lack of sufficient or appropriate information from healthcare professionals, often feeling they were left to look online themselves. For many ICs, this was compounded by challenges with health literacy, as not knowing what to expect often meant they did not know what knowledge was required to effectively advocate for the care recipient’s healthcare. Some participants detailed instances of frustration where the care recipient did not welcome the support they provided, or where support was (or would be) resisted (eg, not wanting to use memory strategies).

### Maintaining the Care Recipient’s Independence

Most ICs spoke about the challenge of finding the balance between providing enough support, while trying not to do too much, to avoid limiting the care recipient’s independence. This was interwoven across all other themes; when providing different types of support, ICs reported the planning and strategies they used to maintain the care recipient’s autonomy (eg, communicating desired support, and using a calendar). Most ICs acknowledged the need to be led by the care recipient, and respect their desired level of support, without too much “fussing.” Still, some participants reported that facilitating independence conflicted with the desire to “care for him as best I can.” This was especially difficult in situations where it would be easier for the IC to just do something themselves for the care recipient (eg, housework). In instances where the care recipient’s support needs decreased over time, many participants reported attempts to cede some responsibilities to the care recipient (eg, walking the dog and housework) and return to “normal,” being mindful not to cause unnecessary stress for the care recipient. Some ICs described challenges with having to “take a back seat” when symptoms improved, after providing support for an extended period.

## Discussion

Partners, family members, and friends, often assume the role of IC to help people with LGG to manage their illness. The supportive role and responsibilities of ICs for people with LGG have not been well investigated, with available literature largely focused on ICs of people with HGG. Our study, therefore, explored how ICs experience the role and responsibilities of supporting someone with an LGG, a group who have (sometimes much) longer prognoses and who can live with multiple, often co-occurring, symptoms and impairments.

ICs in our study reported their experiences with providing cognitive, emotional, and practical support, and being a healthcare advocate; the level of care provided varied across participants. The themes in our findings reflect several aspects of the social support and stress buffer hypothesis,^31^ indicating that there are different types of social support that people provide. The breadth of support provided emphasizes the importance of ICs in helping people with LGG to manage their illness. Echoing this, in another part of the Ways Ahead project, which involved interviews with people with LGG, “receiving support from family and friends” was the most common self-management strategy reported.^32^ The types of support reported in the present study largely reflect what is known about ICs’ responsibilities from other brain tumor studies^18^; still, the paucity of evidence specifically focused on ICs of people with LGG means this study brings value in highlighting that the caregiving responsibilities known for ICs of people with HGG are also applicable to this population. The importance of this study is, therefore, in ensuring that the responsibilities and potential support needs of ICs of people with LGG are not overlooked.

Where this study provides unique insight is with the cross-cutting theme related to “Maintaining the care recipient’s independence,” which was interwoven across all other themes. Specifically, it was noteworthy that ICs often described challenges around trying not to do too much, particularly with the management of medication, health appointments, and finances. While these were perceived as important issues to “manage,” ICs were often conscious of maintaining the care recipient’s autonomy, acknowledging that taking too much control over these matters could have consequences for the independence of the care recipient. This appears to be a distinct challenge for ICs of people with LGG, compared to people with HGG, who have higher physical dependency and require more direct care.^19,21^ Still, our findings suggest that ICs may sometimes lack confidence in the care recipient’s ability to make important decisions. Healthcare providers can assess an individual’s mental capacity to make a specific decision, if concerns are raised about their decision-making ability.^33^ This is important because we have reported elsewhere from the interviews with people with LGG, that excessive or unsolicited support limited care recipients’ independence and created a barrier to self-management.^34^

In terms of supporting participation in daily life, our findings go beyond previous caregiving studies in other cancers^10,35^ and neurological conditions^36,37^ to highlight how ICs may prioritize the needs of the care recipient over their own; for example, assuming increased household/childcare responsibilities to ensure the care recipient could preserve their energy for social activities. Moreover, ICs outlined the strategies they implemented to mitigate risk and distress for the care recipient, such as introducing index cards to adjust for cognitive difficulties. However, we also show that the care recipient needs to be willing to engage with the strategy, which could be influenced by tumor-related behavioral and personality changes (eg, lack of motivation or initiative).^38^ This has further implications for whether a collaborative relationship dynamic can be achieved, that does not restrict the independence of the care recipient.^8^ It may be valuable for healthcare providers to support, as part of rehabilitation, the *co-development* of acceptable self-management strategies with people with LGG and their ICs; these could be goal focused and work towards greater independence for the person with LGG, in turn potentially reducing carer load for the IC. We would endorse the calls from others for research into the effectiveness of cognitive rehabilitation in supporting the autonomy of people with brain tumors^39^; interventions in other contexts (eg, primary progressive aphasia) show the value of this, for example, rehabilitation to support ICs as communication partners.^40^

The provision of “emotional protection” was a strong theme in this analysis and is consistent with the wider caregiving literature in other cancers^7,9,35^ and neurological conditions.^36,37^ However, the incurable nature of LGGs may present a distinct challenge. For example, it was striking that our participants spoke about avoiding expressing negative emotions; while this may be done with the intention of “protecting” the care recipient, it might also reflect ICs’ attempts to “protect” themselves, with avoidance of communication around “difficult” issues associated with anticipatory grief.^41,42^ ICs in our study focused on ensuring the care recipient felt supported, through desired levels of companionship; this was, however, underpinned by the fact that it was sometimes difficult to “be there” for the care recipient, either due to not living with them or conflict with work responsibilities. While some participants described moving from full-time to part-time working to facilitate caregiving, we also found that ICs often felt responsible for alleviating financial pressures, where the care recipient was unable to work. This echoes how family resources adapt in families of people with brain tumors,^43^ and suggests that the financial impact of LGG on families is worthy of further exploration.

The role transition to “Being a ‘carer’” in our findings is consistent with other qualitative studies in ICs of people with brain tumors.^18^ Still, the potential for a long-term prognosis in people with LGG means that the assumed role and responsibilities need to be sustainable. Here, we acknowledge that one-off interviews capture only a “snapshot” of experiences at a single timepoint; we also did not explicitly capture the time since the care recipient’s diagnosis. Therefore, this study does not shed light on the trajectory of the caring role and responsibilities over time, whether or how this varies, and what might impact that trajectory. This is important because the breadth of support reported by our participants outlines where people with LGG may struggle if they do not have that support available within their informal networks. This warrants further investigation to highlight both, the experiences of people with LGG with weaker support networks, and timepoints across the illness trajectory when additional support may be required.

In earlier analyses of this dataset, we reported the constellation of emotional impacts experienced by these ICs,^27^ and highlighted how ICs themselves benefit from a broad range of support (eg, opportunities for relief and opportunities to talk) to help them manage and adapt to their caregiving role.^44^ To add to this, here, participants emotively described feeling “abandoned” by the healthcare system and reported that this was compounded by a lack of respite once they had made the shift to become carers. Moreover, our participants frequently reported challenges with a lack of, or difficulty finding, sufficient and appropriate information. Research in other cancers shows that ICs’ unmet healthcare service needs could be related with decreased quality-of-life in ICs^45^ and negatively impact the well-being of the care recipient.^46^ Given that ICs of people with brain tumors report poor quality-of-life,^47^ our findings and the issue of long-term sustainability accentuate the importance of finding ways to meet ICs’ support needs. For example, ICs may benefit from stronger connections with healthcare professionals,^48^ including integration, where possible, in the dissemination of advice and signposting to support.^49^ Nonetheless, to ensure that care remains person-centered for people with LGG, such integration of ICs needs to be appropriately managed and align with the desires and priorities of the care recipient.

The practical insights from this study which could help better meet the needs of ICs are summarized in the box. Of course, it is important not to divorce the perspective of the care recipients from that of the ICs; while there was no data in our interviews with people with LGG that indicate they would not be open to these suggestions,^32,34^ the care recipient’s needs must be paramount. Therefore, careful consideration is needed of how to ensure their autonomy is maintained in implementing strategies to better support ICs.

**Summary of practical insights from this study**

- During the care recipient’s rehabilitation, there is a need to co-develop acceptable, goal-focused self-management strategies that support the autonomy of people with LGG.
- There is a need for appropriately managed integration of ICs in the dissemination of information and support to facilitate the sustainable fulfillment of their caregiving role and responsibilities.
- When developing supportive care plans, there is a need to acknowledge how the support needs of each person with LGG may be influenced by the strength of their informal network.

### Strengths and Limitations

Our findings are supported by multiple quotes; hence, we are confident that we generated sufficient data to understand how ICs experience the role and responsibilities of supporting someone with an LGG, who, to date, are an under-investigated study population. Our sample largely included spousal ICs, with the few non-spousal ICs often not living with the care recipient; the specific challenges experienced by this group would be worthy of further exploration. Although this analysis focused entirely on the responsibilities of the IC, we acknowledge that caregiving may not be one directional, and that the care recipient may also provide support to the so-called IC; this dynamic should be explored in future research. Due to Covid-19, most of our recruitment was via the Brain Tumour Charity, which may mean that we recruited ICs that have adopted a more “active” support role and a particular perspective on some issues. While we asked each participant for some information about the care recipient (eg, tumor type), we did not formally record this, so cannot report this here. Finally, some of the reported challenges (eg, regarding financial support and healthcare advocacy) may not be entirely transferable, as they may be somewhat dependent on the UK’s healthcare, social welfare, and legal systems. For example, NHS healthcare is free at the point of delivery; in settings where this is different (eg, privatized healthcare in the United States), ICs may encounter different challenges, for example, with healthcare insurance. Nonetheless, the breadth of our findings means we still add substantial value to the limited evidence base.

## Conclusions

This study explored how ICs experience the role and responsibilities of supporting people with LGG. ICs in our study offered wide-ranging support to help manage the consequences of the illness, emphasizing the value of their supportive role. However, the provision of care was underpinned by several challenges, particularly related to balancing support provision without inhibiting the care recipient’s independence, and the need for information and support from healthcare services. Consideration of ways to help ICs manage the challenges faced is needed to facilitate the fulfillment of their supportive role, which could, in turn, help improve outcomes for ICs and people with LGG.

## Supplementary material

Supplementary material is available online at *Neuro-Oncology Practice* (https://academic.oup.com/nop/).

npae096_suppl_Supplementary_File
